# The Plumber Versus Electrician Dilemma: To Bypass or to Defibrillate?

**DOI:** 10.7759/cureus.58543

**Published:** 2024-04-18

**Authors:** Sravani Singu, Brian D Dearing, Robert P Robichaux, Jr., Ralph S Buckley

**Affiliations:** 1 Internal Medicine, Thomas Hospital, Infirmary Health, Fairhope, USA; 2 Clinical and Interventional Cardiology, Cardiology Associates, Fairhope, USA; 3 Cardiac Electrophysiology, Cardiology Associates, Fairhope, USA; 4 Clinical and Non-Invasive Cardiology, Cardiology Associates, Fairhope, USA

**Keywords:** polymorphic ventricular tachycardia, cardiac arrest, coronary artery bypass graft, coronary artery disease, implantable cardioverter-defibrillator, syncope

## Abstract

We present a unique case of an 80-year-old male who presented to our emergency department following cardiac defibrillation when he was found to be in polymorphic ventricular tachycardia (VT) after a syncopal event while at cardiac rehabilitation. He had known coronary artery disease and had a four-vessel coronary artery bypass graft (CABG) 20 years prior to presentation. He underwent left heart catheterization (LHC) two months prior to the syncopal event for worsening shortness of breath and the decision at that time was to proceed with medical management and intervene with redo-CABG if shortness of breath did not improve or progressively worsened. While admitted under our care after the polymorphic VT event, we faced the dilemma of whether to proceed with redo-CABG first since cardiac ischemia is a common cause of polymorphic VT or whether to insert an implantable cardioverter-defibrillator (ICD) before proceeding with redo-CABG. We present the current literature that addresses ICD implantation for secondary prevention and our approach to this complicated case.

## Introduction

Sudden cardiac death (SCD) accounts for 15-20% of mortality globally and is frequently caused by ventricular arrhythmias [1]. In the Western world, coronary heart disease is the underlying cause of ventricular arrhythmias leading to SCD. Implantable cardioverter-defibrillator (ICD) is recommended per guidelines for those who have survived cardiac arrest attributable to ventricular tachycardia (VT), ventricular fibrillation (VF), or those who have sustained VT not due to a reversible cause and have greater than a one-year life expectancy [1]. Three landmark randomized clinical trials Cardiac Arrest Study Hamburg (CASH), Anti-arrhythmic Versus Implantable Defibrillator (AVID), and Canadian Implantable Defibrillator Study (CIDS) evaluating the efficacy of ICD therapy thus far have shown that ICD is highly effective, well tolerated, and cost-effective when compared with conventional drug therapy [2]. ICD is now considered first-line therapy for secondary prevention of VT/VF. Patients with reversible etiology of arrhythmia (i.e., acute ischemia, electrolyte abnormalities) were excluded from the above trials, but they remain at increased risk of arrhythmic and all-cause mortality [3]. We present a patient case in which we concluded that acute ischemia led to polymorphic VT causing cardiac arrest and explain the dilemma of whether to proceed initially with revascularization to treat what we believe was the underlying cause of the arrhythmia or to place an ICD to prevent further arrhythmic events and then proceed with revascularization.

## Case presentation

**Figure 1:**
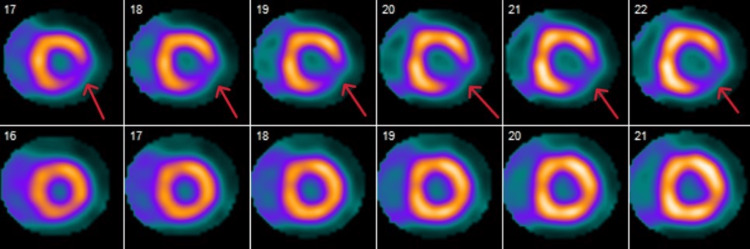
Basal short-axis view of PET CT with arrows pointing to areas of ischemia in the basal inferolateral region consistent with mid-left circumflex and obtuse marginal 1 artery occlusion.

**Figure 2:**
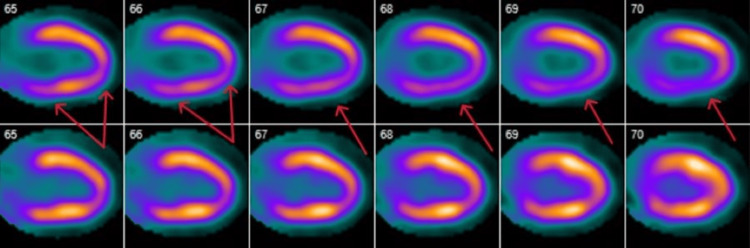
Vertical long-axis view of PET CT with arrows pointing to areas of ischemia in the apical region consistent with prior total left anterior descending artery occlusion.

An 80-year-old male presented to our emergency department following cardiac defibrillation when he was found to be in polymorphic VT after a syncopal event while in cardiac rehabilitation. Cardiopulmonary resuscitation (CPR) was started immediately and return of spontaneous circulation (ROSC) was achieved within three minutes. Medical history is significant for coronary artery disease with prior four-vessel coronary artery bypass graft (CABG), hypertension, and hyperlipidemia. The patient had seen a cardiologist for shortness of breath three months prior to this hospitalization. At that time, a positron emission tomography (PET) scan and transthoracic echocardiogram (TTE) were obtained. The PET scan showed inferolateral segment ischemia with a large severe reversible defect from base to apex and preserved left ventricular ejection fraction of 59% at rest and 61% with stress (Figures 1, 2). 

TTE showed a left ventricular ejection fraction (LVEF) of 50 to 55% and no significant valvular disease nor wall motion abnormalities were noted (Video 1). The patient underwent left heart catheterization (LHC) in January 2024, and it showed 75 to 80% stenosis of the left main coronary artery and proximal total occlusion of the left anterior descending artery (LAD) with patent left internal mammary artery graft to the LAD. The mid circumflex was occluded and obtuse marginal 1 (OM1) was subtotally occluded with good collateral filling from the diagonals. The mid-right coronary artery (RCA) had complete total occlusion (CTO) with a patent vein graft to the posterior descending artery (PDA). Balloon angioplasty was done for the left main artery, however, the OM1 was unable to be revascularized. After the LHC, the patient was medically managed with low-dose aspirin, clopidogrel, lisinopril, metoprolol succinate, isosorbide mononitrate, high-dose atorvastatin, and as-needed sublingual nitroglycerin. The patient had voiced understanding with the plan to go forward with medical management and proceed with redo-CABG if shortness of breath had persisted or worsened. 

**Video 1:** Transthoracic echocardiogram showing left ventricular ejection fraction of 50-55%, no wall motion abnormalities, and no significant valvular disease three months prior to the ventricular arrhythmia event.

A review of systems was positive for a two-year history of shortness of breath that worsens with exertion and weakness. He denied chest pain, palpitations, lower extremity edema, orthopnea, and paroxysmal nocturnal dyspnea. He is a former smoker, who quit smoking two decades prior. He drank alcohol socially and had never used illicit drugs. 

**Figure 3:**
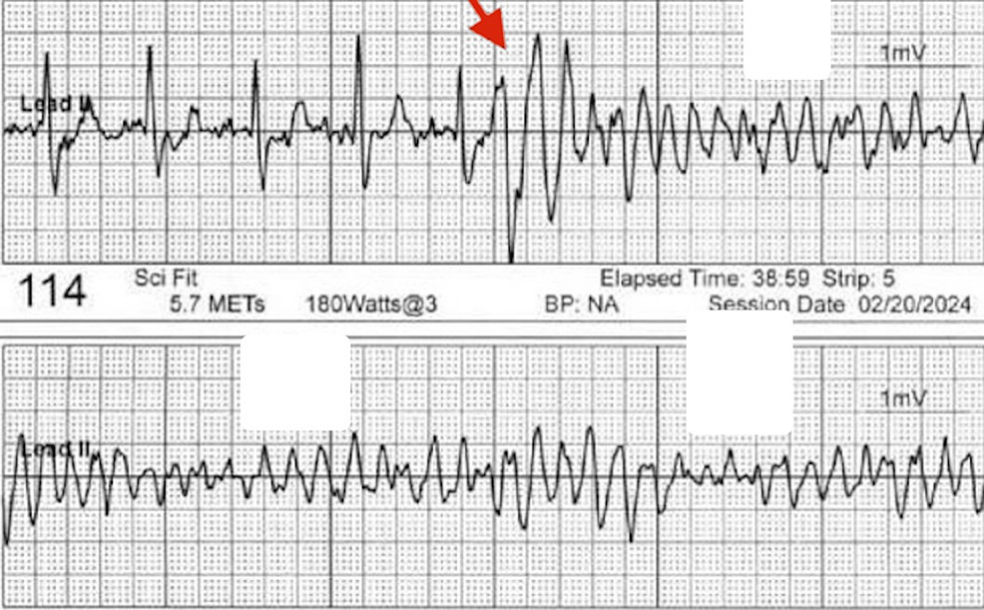
The red arrow in the top rhythm strip shows the onset of polymorphic ventricular tachycardia. The bottom rhythm strip shows the continuation of polymorphic ventricular tachycardia.

Vital signs on presentation were: blood pressure 103/80 mmHg, heart rate 68 beats per minute, respiratory rate 18 breaths per minute, temperature 98.3 degrees Fahrenheit, oxygen saturation 95% on room air. Complete blood count (CBC) and comprehensive metabolic panel (CMP) with magnesium were within normal limits. High sensitivity troponin was elevated initially and then trended upward. Telemetry strips during the polymorphic VT events are shown in Figure 3. An electrocardiogram (ECG) after defibrillation showed anteroseptal and anterolateral ST segment depressions and a chronic right bundle branch block. Chest x-ray was unremarkable. TTE showed LVEF of 45-50% without significant valvular disease or wall motion abnormalities. On physical examination, the patient was not in acute distress, had regular heart rate and rhythm, lungs were clear to auscultation bilaterally, had trace bilateral pedal edema, and had right supraorbital and maxillary abrasions from the syncopal event. All other systems were unremarkable on the physical exam.

We suspected that the patient converted into polymorphic VT due to coronary ischemia, the most common cause of the arrhythmia, especially given his history of coronary artery disease and having had bypass surgery previously. Cardiology, cardiothoracic surgery, and electrophysiology teams were consulted. The dilemma between the two specialists became whether to revascularize the coronaries with a redo-CABG or whether to place an ICD initially. After discussing current guidelines, we opted to proceed initially with the redo-CABG with the reasoning that the underlying cause of the ischemia leading to arrhythmias should be treated first and may lower the immediate risk of further arrhythmias. The patient underwent a one-vessel bypass of the OM1. He did well post-CABG and given his excellent recovery, a dual chamber ICD was placed one week later for secondary prevention. The patient has since followed up in the cardiology clinic and has been doing well postoperatively with neither VT nor VF events. 

## Discussion

SCD accounts for 15-20% of mortality globally and is frequently caused by ventricular arrythmias. In the Western world, coronary ischemia is the underlying cause of ventricular arrhythmias leading to 50% of SCD events [1]. ICD therapy is recommended for secondary prevention per guidelines for those who have survived cardiac arrest attributable to VT, VF, or those who have sustained VT not due to a reversible cause and have greater than a one-year life expectancy [1]. Three landmark trials Cardiac Arrest Study Hamburg (CASH), Antiarrhythmics Versus Implantable Defibrillator Study (AVID), and Canadian Implantable Defibrillator Study (CIDS) evaluated the efficacy of ICD therapy for secondary prevention of sudden cardiac death [2,3,4]. The CASH trial studied the efficacy of ICD vs medical therapy with either amiodarone or metoprolol in survivors of cardiac arrest due to either VT or VF. It found that the ICD significantly reduced mortality by 37% compared to medical therapy [1]. There was no significant difference between amiodarone and metoprolol when compared with the ICD arm. The AVID trial evaluated the difference between ICD and medical therapy with either amiodarone or sotalol in those who were resuscitated after VF arrest, sustained VT with syncope, or sustained VT with LVEF less than or equal to 40% and signs and symptoms of hemodynamic compromise (chest pain, hypotension, near-syncope). The study was terminated early due to the ICD arm demonstrating a significantly decreased death rate when compared to medical therapy with amiodarone. The CIDS trial also compared ICD versus amiodarone therapy and did not result in a statistically significant reduction in all-cause mortality between the two arms due to insufficient statistical power [1]. 

Randomized clinical trials thus far have shown that ICD therapy is highly effective, well-tolerated, and cost-effective when compared with conventional drug therapy. ICD, therefore, is now considered first-line therapy for secondary prevention of VT and VF [1]. Patients with reversible etiologies of arrhythmia (i.e., acute ischemia, electrolyte or metabolic abnormalities, use of proarrhythmic medications) were excluded from the above trials, however, they remain at increased risk of arrhythmic and all-cause mortality [5]. Based on the principle that there is a low recurrent rate of ventricular arrhythmias if the underlying reversible etiology is treated, SCD survivors with a reversible cause are deemed ineligible for ICD implantation [5]. A systematic review conducted by Van der Lingen et al. analyzed studies evaluating ICD implantation in SCD survivors with reversible etiologies, which were divided into four subgroups: acute myocardial infarction, coronary artery spasm, takotsubo cardiomyopathy, and various other causes of cardiac arrest [5]. They focused on two primary outcomes, mortality and appropriate device therapy, which they defined as anti-tachycardia pacing or ICD shock for sustained VT or VF. They found that most of the studies they analyzed reported high rates of appropriate device therapy. Unfortunately, the high rates of appropriate device therapy do not translate into a survival benefit. The data on the mortality benefit of treatment with ICD after SCD survival due to acute MI is inconsistent according to the systemic review. Some studies reported a decrease in mortality while some reported no difference between those with and without ICD. One study even reported a higher mortality in those with ICD implantation. One limitation of the systematic review by Van der Lingen et al. is that the studies they analyzed lacked a randomized design [5]. By presenting our case and the dilemma we faced along with the inconsistent prior study results, we emphasize that there is a need to conduct further research into the outcomes of ICD implantation in cases of underlying ischemic etiologies.

## Conclusions

We presented a case in which our patient nearly succumbed to sudden cardiac death (SCD) due to ventricular tachycardia (VT) from ischemic cardiomyopathy. Due to unclear literature on whether there is a benefit from implantable cardioverter-defibrillator (ICD) implantation in SCD survivors due to VT or ventricular fibrillation from ischemic etiologies, we came across the dilemma of whether to perform revascularization therapy followed by ICD implantation or vice versa in our patient. It is important to recognize that myocardial scar from ischemic events can precipitate ventricular arrhythmias by creating re-entry circuits. Therefore, regardless of revascularization therapy, these patients remain at high risk for ventricular arrhythmias and will benefit from ICD implantation following revascularization. We emphasize the need to conduct further research into the outcomes of ICD implantation in cases of underlying ischemic etiologies.
